# New generalized Trends of Coupled Fixed Point Theorems for Continuous Mappings Satisfying a Property of Mixed Monotone

**DOI:** 10.12688/f1000research.172256.1

**Published:** 2025-12-05

**Authors:** Mustafa K. Bardan, Alaa M.F. Al-Jumaili

**Affiliations:** 1 Department of mathematics, College of Education for pure sciences, University of Anbar, Ramadi, Anbar, 31001, Iraq

**Keywords:** D^*-metric spaces; partially ordered D^*-metric spaces; D^*-complete metric; coupled fixed point; property of mixed monotone; partially ordered set; D^*-continuous maps.

## Abstract

**Background:**

One of the most important results of mathematical analysis is the Banach fixed point theory, he explained in this theory that a mapping T: X → X always has a unique fixed point in X. After witnessing the implementations of this theory in giving the existence and uniqueness solutions for many integral and differential equations, additionally discovery of solutions for linear and nonlinear systems, various extensions of this theory were carried out. Our main results represent one of the most important of these generalizations in the literature.

**Methods and Results:**

Various vital concepts are needed in the sequels which are playing a major role in verifying our major outcomes have been presented. Throughout this manuscript, (X,≼) indicates to a partially ordered set with the partially ordered ≼ . In this study, our main objective is to investigate and verify various new enhanced results of coupled fixed point theorems for continuous maps having the property of mixed monotone under the influence of extended contraction circumstances in the context of partially ordered $D^{*}$ -complete metric spaces. Numerous characterizations of these types of coupled fixed point theorems have been verified. Our major outcomes are extending and enhancing the various outcomes of coupled fixed point theorems existing in the literature. Additionally, an appropriate example that supports the major outcomes was prepared.

**Conclusions:**

Our main results in this manuscript have been explored novel various outcomes related to the uniqueness of various coupled fixed point theorems for continuous maps having the property of mixed monotone under the influence of extended contraction circumstances in the context of partially ordered $D^{*}$ -complete metric spaces. We predict that the discoveries in this study will aid scientists in enhancing the research on popularized partially ordered metric spaces to elevate a universal framework for their practical implementations.

## 1. Introduction

The idea of coupled fixed point theory is an extremely active branch of mathematics and is at the essence of nonlinear analysis because it provides an influential tool to confirm the existence and uniqueness of solutions for numerous nonlinear issues emerging with pure and applied mathematics and other branches of sciences, such as computer science, engineering, physics, differential equations, optimization, control theory, approximation theory, and discrete dynamics. One of the most important results of mathematical analysis is the Banach contraction mapping. It is a popular tool for solving existing issues in different fields of pure and applied mathematics. Numerous extensions of Banach contraction mapping have been proposed via some authors in the literature, such as. A, Ran, and M. Reurings ^1^ extended Banach contraction mapping in a partially ordered set with various implementations to linear and nonlinear matrix equations. Nieto and R. López ^2^ generalized the results of the authors in ^1^ and utilized their major outcomes to obtain a unique solution concerning satisfactory first-order differential periodic boundary circumstances. Subsequently, the notions of coupled fixed point and mixed monotone mappings were presented by T. Bhaskar and V. Lakshmikantham, ^3^ and several coupled fixed points resulted in partially ordered metric space, in addition to utilizing their outcomes on a first-order ordinary differential with periodic boundary circumstances. Presently, Shaban et al. ^4^ verified the idea $D^{*}$ -metric spaces, which are extensions of ordinary metric spaces. However, after the publication of this work, numerous results related to coupled, common coupled, and coupled coincidence fixed points have been reported in the literature. ^5– 9^ Motivated by these facts, Shah et al. ^10^ investigated and proved various coupled fixed point theorems for integral-type contractive mappings in G-metric spaces. Furthermore, T. Oklah and A. Al-Jumaili ^11^ employed the notion of compatibility for hybrid pairs of mappings and verified several common coupled coincidence fixed point outcomes with satisfactory properties of mixed G -monotone in partially ordered $D^{*}$ -metric spaces. Additionally, in the same year N. A. Majid, et al. ^12^ verified several novel outcomes of fixed points for monotone multi-valued maps in partially ordered $D^{*}$ -complete metric spaces, and other various results of coupled fixed point of maps satisfying contractive conditions have been obtained, as well ^13^ they discussed and confirmed various outcomes of common and coincidence of fixed points theorems in S-complete metric spaces. Recently, Oklah and Al-Jumaili ^14^ offered and investigated some practical implementations for pairs of self maps satisfy extended contractive conditions of the integral kind in $D^{*}$ -metric spaces and obtained some common and coupled fixed point results in such spaces. For future studies, we can expand and generalize our results to other spaces, such as. ^15– 18^ The inspiration for introducing this manuscript is to consider and verify novel extended categories of coupled fixed point outcomes for continuous maps satisfying the properties of mixed monotone under the influence of generalized contraction circumstances in context of partially $D^{*}$ -complete metric spaces. In addition, introduce an appropriate example to support our main results. Our major outcomes, which are related to these categories of coupled fixed points, generalize and improve the various results existing in the literature.

## 2. Materials and methods

This section is devoted to remembering various ideas and significant outcomes that play a vital role in this work and to confirming our major outcomes. Throughout this manuscript, (X,≼) indicates to a partially ordered set with the partially ordered ≼ . Via $y^{*}≺\varkappa^{*}$ holds, mean $y^{*}≼\varkappa^{*}$ holds, and via $\varkappa^{*}≺y^{*}\;$ holds, mean $\varkappa^{*}≼y^{*}$ holds, with $\varkappa^{*}\neq y^{*}$ .

Definition 2.1: ^4^ Suppose that $D^{*}:X^{3}\to {\mathbb{R}}^{+}$ , is a mapping described on X≠∅ and satisfactory the next conditions $\forall \varkappa^{*},y^{*},z^{*},w^{*}\in X$ :

$\left(D_{1}^{*}\right)$ $D^{*}$ $\left(\varkappa^{*},y^{*},z^{*}\right)\geq 0,\forall \varkappa^{*},y^{*},z^{*}\in X$ ;

$\left(D_{2}^{*}\right)$ $D^{*}$ $\left(\varkappa^{*},y^{*},z^{*}\right)=0$ *iff* $\varkappa^{*}=y^{*}=z^{*}$ ;

$\left(D_{3}^{*}\right)$ $D^{*}$ $\left(\varkappa^{*},y^{*},z^{*}\right)=D^{*}\left(P\left\{\varkappa^{*},y^{*},z^{*}\right\}\right),$ (Symmetry) where P is permutation map,

$\left(D_{4}^{*}\right)$ $D^{*}$ $\left(\varkappa^{*},y^{*},z^{*}\right)\leq D^{*}\left(\varkappa^{*},y^{*},w^{*}\right)+$ $D^{*}$ $\left(w^{*},z^{*},z^{*}\right)$ .

So, $D^{*}$ is called $\;D^{*}$ -metric and $\left(X,D^{*}\right)$ called $D^{*}$ -metric space (Concisely, $D^{*}$ -M-sp).

Example 2.2: ^4^ Lineal examples of such a map are:

(i)
$D^{*}\left(\varkappa^{*},y^{*},z^{*}\right)=\max\left\{|\varkappa^{*}-y^{*}|,|y^{*}-z^{*}|,|\varkappa^{*}-z^{*}|\right\}$ ,
(ii)
$D^{*}$ $\left(\varkappa^{*},y^{*},z^{*}\right)=\left|\varkappa^{*}-y^{*}\right|+\left|y^{*}-z^{*}\right|+\left|\varkappa^{*}-z^{*}\right|$ .
(iii)
IfX=ℝ. So describe: $D^{*}\left(\varkappa^{*},y^{*},z^{*}\right)=\left\{ \begin{array}{ll} 0 & \text{if}\;\varkappa^{*}=y^{*}=z^{*}\;\\ \max\left\{\varkappa^{*},y^{*},z^{*}\right\} & \text{otherwise}\; \end{array}\right.$

Definition 2.3: ^4^ Presume that $\left(X,D^{*}\right)$ is a $D^{*}$ - M-sp; therefore,

(i) A sequence $\left\{\varkappa_{s}^{*}\right\}$ is called converges to $\varkappa^{*}\in X$ *iff* $D^{*}\left(\varkappa_{s}^{*},\varkappa_{s}^{*},\varkappa^{*}\right)=D^{*}\left(\varkappa^{*},\varkappa^{*},\varkappa_{s}^{*}\right)\to 0\;\text{as}\;s\to \infty$ . i.e., equivalent with, $\forall \varepsilon >0,\exists ℯ\in {\mathbb{Z}}^{+}$ (s. t), $\forall s,r\geq ℯ⟹D^{*}\left(\varkappa^{*},\varkappa_{s}^{*},\varkappa_{r}^{*}\right)<\varepsilon$ .
(ii) A sequence $\left\{\varkappa_{s}^{*}\right\}$ is called $D^{*}$ -Cauchy if $\forall \varepsilon >0,\exists ℯ\in {\mathbb{Z}}^{+}$ ; $\forall s,r\geq ℯ,D^{*}\left(\varkappa_{s}^{*},\varkappa_{s}^{*},\varkappa_{r}^{*}\right)<\varepsilon$ . That is, if $\;D^{*}\left(\varkappa_{s}^{*},\varkappa_{s}^{*},\varkappa_{r}^{*}\right)\to 0\;\mathrm{as},s,r\to \infty$ .
(iii)
$\left(X,D^{*}\right)$ is called $D^{*}$ -complete M-sp. If each $D^{*}$ -Cauchy sequence is convergent in X .

Remark 2.4: In Ref. 4, it has been illustrated that $D^{*}$ -M-sp induces the Housdorff topology with convergence, as demonstrated in  Def-2.3, relative to this kind of topology. This topology is Housdorff, with $\left\{\varkappa_{s}^{*}\right\}$ converge to only one point at most.

Proposition 2.5: ^19^ The next statements are equivalent in $\left(X,D^{*}\right)$ :

(i)
$\left\{\varkappa_{s}^{*}\right\}$ is $D^{*}$ -convergent to $\varkappa^{*};$

(ii)
$D^{*}\left(\varkappa_{s}^{*},\varkappa^{*},\varkappa^{*}\right)\to 0$ , as (s→∞);

(iii)
$D^{*}\left(\varkappa_{s}^{*},\varkappa_{s}^{*},\varkappa^{*}\right)\to 0$ , as (s→∞);

Lemma 2.6: ^4^ Next statements are equivalent in $\left(X,D^{*}\right):$

(i) The sequence $\left\{\varkappa_{s}^{*}\right\}$ is $D^{*}$ -Cauchy;
(ii)
$\forall \varepsilon >0,\exists ℯ\in {\mathbb{Z}}^{+}$ (s. t), $D^{*}\left(\varkappa_{s}^{*},\varkappa_{s}^{*},\varkappa_{r}^{*}\right)<\varepsilon \forall s,r\geq ℯ$ .

Lemma 2.7: ^4^ If $\left(X,D^{*}\right)$ is $D^{*}$ -M-sp, thus $D^{*}\left(\varkappa^{*},\varkappa^{*},y^{*}\right)\leq 2D^{*}\left(y^{*},y^{*},\varkappa^{*}\right)\forall \varkappa^{*},y^{*}\in X$ .

By combining Lemma 2.6 and Lemma 2.7 we obtain the next outcome:

Lemma 2.8: If $\left(X,D^{*}\right)$ is $D^{*}$ -M-sp, hence $\left\{\varkappa_{s}^{*}\right\}$ is $D^{*}$ -Cauchy *iff* $\forall \varepsilon >0,\exists ℯ\in {\mathbb{Z}}^{+}$ where, $D^{*}\left(\varkappa_{s}^{*},\varkappa_{s}^{*},\varkappa_{r}^{*}\right)<\varepsilon \forall s>r\geq ℯ$ .

Definition 2.9: ^4^ $\left(X,D^{*}\right)$ called symmetric if $D^{*}\left(\varkappa^{*},\varkappa^{*},y^{*}\right)=$ $D^{*}$ $\left(\varkappa^{*},y^{*},y^{*}\right),$ $\forall \varkappa^{*},y^{*}\in X$ , As well $\left(X,D^{*}\right)$ is said to be non-Symmetric if it’s not-Symmetric.

Lemma 2.10: ^4^ Each $D^{*}$ -metric on X induces a metric $d_{D^{*}}$ on X via

$d_{D^{*}}\left(\varkappa^{*},y^{*}\right)=$ $D^{*}$ $\left(\varkappa^{*},y^{*},y^{*}\right)+$ $D^{*}$ $\left(y^{*},\varkappa^{*},\varkappa^{*}\right),\forall \varkappa^{*},y^{*}\in X$ , for $D^{*}$ -Symmetric

$d_{D^{*}}\left(\varkappa^{*},y^{*}\right)=2D^{*}\left(\varkappa^{*},y^{*},y^{*}\right),\forall \varkappa^{*},y^{*}\in X$ .

Example 2.11: Assume that $X={\mathbb{Z}}^{+}$ , describe:

$$D^{*}\left(\varkappa^{*},\varkappa^{*},y^{*}\right)=\left\{ \begin{array}{c} \varkappa^{*}+y^{*}\;\text{if}\;\varkappa^{*}<y^{*}\\ \varkappa^{*}+y^{*}+\frac{1}{2}\;\text{if}\;\varkappa^{*}>y^{*} \end{array}\right.,$$

$$D^{*}\left(\varkappa^{*},y^{*},z^{*}\right)=\left\{ \begin{array}{ll} 0 & \text{if}\;\varkappa^{*}=y^{*}=z^{*}\\ \varkappa^{*}+y^{*}+z^{*} & \text{if}\;\varkappa^{*}\neq y^{*}\neq z^{*}\;\\ \text{with symmetry in each three variables} \end{array}\right.$$

In this case, $\left(X,D^{*}\right)$ is $D^{*}$ -M-sp and non-symmetric, because if $\;\varkappa^{*}<y^{*}$ , obtain

$$D^{*}\left(\varkappa^{*},\varkappa^{*},y^{*}\right)=\varkappa^{*}+y^{*}\neq \varkappa^{*}+y^{*}+\frac{1}{2}=D^{*}\left(\varkappa^{*},y^{*},y^{*}\right)$$

Remark 2.12: If $\left(X,D^{*}\right)$ is non $D^{*}$ -symmetric-sp, the $D^{*}$ -metric properties demonstrate that

$$\frac{3}{2}D^{*}\left(\varkappa^{*},y^{*},y^{*}\right)\leq d_{D^{*}}\left(\varkappa^{*},y^{*}\right)=3D^{*}\left(\varkappa^{*},y^{*},y^{*}\right),\forall \varkappa^{*},y^{*}\in X.$$

Definition 2.13: ^20^ Assume $\left(X,D^{*}\right)$ and $\left(X^{*},D^{**}\right)$ are two $D^{*}$ -M-sp. Then, $F:X⟶X^{*}$ is $D^{*}$ -continuous at $\varkappa^{*}\in X$ *iff* it’s $D^{*}$ -sequentially continuous at $\varkappa^{*}$ ,i.e., when $\left\{\varkappa_{s}^{*}\right\}$ is $D^{*}$ -convergent to $\varkappa^{*}$ , $\left\{F\left(\varkappa_{s}^{*}\right)\right\}$ is $D^{*}$ -convergent to $F\left(\varkappa^{*}\right)$ .

Definition 2.14: ^11^ Let $\left(X,D^{*}\right)$ be $D^{*}$ -M-sp. A map F:X×X⟶X is called continuous if for arbitrary two $D^{*}$ -convergent sequences $\left\{\varkappa_{s}^{*}\;\right\}$ & $\left\{y_{s}^{*}\;\right\}$ converging to $\varkappa^{*}$ & $y^{*}$ correspondingly, $\left\{F\left(\varkappa_{s}^{*},y_{s}^{*}\right)\right\}$ is $D^{*}$ -convergent to $F\left(\varkappa^{*},y^{*}\right)$ .

Definition 2.15: ^3^ Presume that (X,≼) partially ordered set (Concisely, P.O.S). A map F:X×X→X is said to have mixed monotone property (Concisely, M.M.P) *if* $F\left(\varkappa^{*},y^{*}\right)$ is monotone nondecreasing in $\varkappa^{*}$ and is monotone nonincreasing of $y^{*}$ ; that is $\forall \varkappa^{*},y^{*}\in X,$

$$\varkappa_{1}^{*},\varkappa_{2}^{*}\in X,\varkappa_{1}^{*}≼\varkappa_{2}^{*}\;\text{Implies}\;F\left(\varkappa_{1}^{*},y^{*}\right)≼F\left(\varkappa_{2}^{*},y^{*}\right),$$

$$\;y_{1}^{*},y_{2}^{*}\in X,y_{1}^{*}≼y_{2}^{*}\;\text{Implies}\;F\left(\varkappa^{*},y_{2}^{*}\right)≼F\left(\varkappa^{*},y_{1}^{*}\right).$$

Definition 2.16: ^3^ An $\left(\varkappa^{*},y^{*}\right)\in X\times X$ , when X≠∅ , called coupled fixed point (Concisely, C.F.P) of a map F:X×X→X , *if* $F\left(\varkappa^{*},y^{*}\right)=\varkappa^{*}$ & $F\left(\;y^{*},\varkappa^{*}\right)=y^{*}$ .

## 3. Some main results of new generalized categories of coupled fixed point theorems

This section is devoted to investigating and verifying various novel extended categories of coupled fixed point outcomes for continuous maps of the satisfactory properties of mixed monotones under various extended contraction conditions in partially ordered complete $D^{*}$ -M-spaces.

Theorem 3.1: Let $\left(X,D^{*}\right)$ be a $D^{*}$ -complete metric space defined on (P.O.S) (X,≼) . Presume that $F:\left(\;X,D^{*}\right)\times \left(\;X,D^{*}\right)\to \left(X,D^{*}\right)\;$ a continuous mapping containing the (M.M.P). Suppose that ∃k∈[0,1) (s. t) for $\varkappa^{*},y^{*},z^{*},p,q,w^{*}\in X,$ the following inequality holds:

$$D^{*}\left(F\left(\varkappa^{*},y^{*}\right),F\left(p,q\right),F\left(w^{*},z^{*}\right)\right)\leq \frac{k}{2}\left[\;D^{*}\left(\varkappa^{*},p,w^{*}\right)+D^{*}\left(y^{*},q,z^{*}\right)\right]$$ (3.1)

$\forall \varkappa^{*}≽p≽w^{*}\&y^{*}≼q≼z^{*}$ wherever either $\;q\neq z^{*}\;\text{or}\;p\neq w^{*}$ . If $\exists \varkappa_{0}^{*},y_{0}^{*}\in X$ (s. t) $\varkappa_{0}^{*}≼F\left(\varkappa_{0}^{*},y_{0}^{*}\right)\&y_{0}^{*}≽F\left(y_{0}^{*},\varkappa_{0}^{*}\right)$ , so F has (C. F. P) in X .

Proof:
Through the condition of above theorem $\exists \varkappa_{0}^{*},y_{0}^{*}\in X\;$ where $\varkappa_{0}^{*}≼F\left(\varkappa_{0}^{*},y_{0}^{*}\right)$ & $y_{0}^{*}≽F\left(y_{0}^{*},\varkappa_{0}^{*}\right)$ . Describe, $\varkappa_{1}^{*},y_{1}^{*}\in X$ as

$$\;\varkappa_{1}^{*}=F\left(\varkappa_{0}^{*},y_{0}^{*}\right)≽\varkappa_{0}^{*}\&y_{1}^{*}=F\left(y_{0}^{*},\varkappa_{0}^{*}\right)≼y_{0}^{*}.$$

Presume that, $\varkappa_{2}^{*}=F\left(\varkappa_{1}^{*},y_{1}^{*}\right)\&y_{2}^{*}=F\left(y_{1}^{*},\varkappa_{1}^{*}\right)$ , we write

$${Ϝ}^{2}\left(\varkappa_{0}^{*},y_{0}^{*}\right)=Ϝ\left(F\left(\varkappa_{0}^{*},y_{0}^{*}\right),F\left(y_{0}^{*},\varkappa_{0}^{*}\right)\right)=Ϝ\left(\varkappa_{1}^{*},y_{1}^{*}\right)=\varkappa_{2}^{*}$$

And

$$F^{2}\left(y_{0}^{*},\varkappa_{0}^{*}\right)=F(F\left(y_{0}^{*},\varkappa_{0}^{*}\right),F\left(\varkappa_{0}^{*},y_{0}^{*}\right)=F\left(y_{1}^{*},\varkappa_{1}^{*}\right)=y_{2}^{*}.$$

Utilizing, the (M. M. P) of F we obtain,

$$\varkappa_{2}^{*}=F^{2}\left(\varkappa_{0}^{*},y_{0}^{*}\right)=F\left(\varkappa_{1}^{*},y_{1}^{*}\right)≽F\left(\varkappa_{0}^{*},y_{0}^{*}\right)=\varkappa_{1}^{*}≽\varkappa_{0}^{*}$$

And

$$y_{2}^{*}=F^{2}\left(y_{0}^{*},\varkappa_{0}^{*}\right)=F\left(y_{1}^{*},\varkappa_{1}^{*}\right)≼F\left(y_{0}^{*},\varkappa_{0}^{*}\right)=y_{1}^{*}≼y_{0}^{*}.$$

Ongoing the above proceedings we get repeatedly, ∀s≥0 ,

$$\varkappa_{s+1}^{*}=F^{s+1}\left(\varkappa_{0}^{*},y_{0}^{*}\right)=Ϝ\left(F^{s}\left(\varkappa_{0}^{*},y_{0}^{*}\right),F^{s}\left(y_{0}^{*},\varkappa_{0}^{*}\right)\right)$$

And

$$y_{s+1}^{*}=F^{s+1}\left(y_{0}^{*},\varkappa_{0}^{*}\right)=Ϝ\left(F^{s}\left(y_{0}^{*},\varkappa_{0}^{*}\right),F^{s}\left(\varkappa_{0}^{*},y_{0}^{*}\right)\right).$$

In that case, ∀s≥0 ,

$$\;\varkappa_{0}^{*}≼F\left(\varkappa_{0}^{*},y_{0}^{*}\right)=\varkappa_{1}^{*}≼F^{2}\left(\varkappa_{0}^{*},y_{0}^{*}\right)=\varkappa_{2}^{*}≼∙∙∙≼F^{s+1}\left(\varkappa_{0}^{*},y_{0}^{*}\right)=\varkappa_{s+1}^{*}≼∙∙∙$$ (3.2)

With

$$y_{0}^{*}≽F\left(y_{0}^{*},\varkappa_{0}^{*}\right)=y_{1}^{*}≽F^{2}\left(y_{0}^{*},\varkappa_{0}^{*}\right)=y_{2}^{*}≽∙∙∙≽F^{s+1}\left(y_{0}^{*},\varkappa_{0}^{*}\right)=y_{s+1}^{*}≽∙∙∙$$ (3.3)

If $\left(\varkappa_{s+1}^{*},y_{s+1}^{*}\right)=\left(\varkappa_{s}^{*},y_{s}^{*}\right),$ in that case F has (C. F. P), as a result we presume

$\left(\varkappa_{s+1}^{*},y_{s+1}^{*}\right)\neq \left(\varkappa_{s}^{*},y_{s}^{*}\right)$ for each s≥0 , that is, we presume that either

$$\varkappa_{s+1}^{*}=F\left(\varkappa_{s}^{*},y_{s}^{*}\right)\neq \varkappa_{s}^{*}\;\text{or}\;y_{s+1}^{*}=F\left(y_{s}^{*},\varkappa_{s}^{*}\right)\neq y_{s}^{*}.$$

Next, we verify that, ∀s≥0 ,

{D∗(Fs+1(ϰ0∗,y0∗),Fs(ϰ0∗,y0∗),Fs(ϰ0∗,y0∗))≤ks2[D∗(F(ϰ0∗,y0∗),ϰ0∗,ϰ0∗)+D∗(F(y0∗,ϰ0∗),y0∗,y0∗)} (3.4)

And

$$\left\{\begin{array}{c} \;D^{*}\left(F^{s+1}\left(y_{0}^{*},\varkappa_{0}^{*}\right),F^{s}\left(y_{0}^{*},\varkappa_{0}^{*}\right),F^{s}\left(y_{0}^{*},\varkappa_{0}^{*}\right)\right)\leq \\ \frac{k^{s}}{2}[\;D^{*}\left(F\left(y_{0}^{*},\varkappa_{0}^{*}\right),y_{0}^{*},y_{0}^{*}\right)+D^{*}\left(F\left(\varkappa_{0}^{*},y_{0}^{*}\right),\varkappa_{0}^{*},\varkappa_{0}^{*}\right) \end{array}\right\}\;$$ (3.5)

For, s=1 , we obtain

$$\left\{\begin{array}{c} D^{*}\left(F^{2}\left(\varkappa_{0}^{*},y_{0}^{*}\right),F\left(\varkappa_{0}^{*},y_{0}^{*}\right),F\left(\varkappa_{0}^{*},y_{0}^{*}\right)\right)=\\ D^{*}\left(F\left(F\left(\varkappa_{0}^{*},y_{0}^{*}\right),F\left(y_{0}^{*},\varkappa_{0}^{*}\right),F\left(\varkappa_{0}^{*},y_{0}^{*}\right),F\left(\varkappa_{0}^{*},y_{0}^{*}\right)\right)\right)\leq \\ \frac{k}{2}\left[D^{*}\left(F\left(\varkappa_{0}^{*},y_{0}^{*}\right),\varkappa_{0}^{*},\varkappa_{0}^{*}\right)+D^{*}\left(F\left(y_{0}^{*},\varkappa_{0}^{*}\right),y_{0}^{*},y_{0}^{*}\right)\right] \end{array}\right\}$$

Utilizing, inequality (3.1), because $\varkappa_{0}^{*}≼F\left(\varkappa_{0}^{*},y_{0}^{*}\right)\&y_{0}^{*}≽F\left(y_{0}^{*},\varkappa_{0}^{*}\right)$ and since either $\varkappa_{s+1}^{*}=F\left(\varkappa_{s}^{*},y_{s}^{*}\right)\neq \varkappa_{s}^{*}\;$ or $\;y_{s+1}^{*}=F\left(y_{s}^{*},\varkappa_{s}^{*}\right)\neq y_{s}^{*}\forall s$ that is

$$D^{*}\left(\varkappa_{2}^{*},\varkappa_{1}^{*},\varkappa_{1}^{*}\right)\leq \frac{k}{2}\left[\;D^{*}\left(\varkappa_{1}^{*},\varkappa_{0}^{*},\varkappa_{0}^{*}\right)+D^{*}\left(y_{1}^{*},y_{0}^{*},y_{0}^{*}\right)\right].$$

Likewise, establish

$$D^{*}\;\left(y_{2}^{*},y_{1}^{*},y_{1}^{*}\right)\leq \frac{k}{2}\left[D^{*}\left(y_{1}^{*},y_{0}^{*},y_{0}^{*}\right)+D^{*}\left(\varkappa_{1}^{*},\varkappa_{0}^{*},\varkappa_{0}^{*}\right)\right].$$

Consequently inequalities (3.4) & (3.5) hold for s=1 .

Next, presume that inequalities- (3.4) & (3.5) hold, for s=r .

Utilizing the truths $F^{r+1}\left(\varkappa_{0}^{*},y_{0}^{*}\right)≽F^{r}\left(\varkappa_{0}^{*},y_{0}^{*}\right)\&F^{r+1}\left(y_{0}^{*},\varkappa_{0}^{*}\right)≼F^{r}\left(y_{0}^{*},\varkappa_{0}^{*}\right)\;$ , we obtain

$$\left\{\begin{array}{c} D^{*}(F^{r+2}\left(\varkappa_{0}^{*},y_{0}^{*}\right),F^{r+1}\left(\varkappa_{0}^{*},y_{0}^{*}\right),F^{r+1}\left(\varkappa_{0}^{*},y_{0}^{*}\right))=\\ D^{*}\left[F\left(F^{r+1}\left(\varkappa_{0}^{*},y_{0}^{*}\right),F^{r+1}\left(y_{0}^{*},\varkappa_{0}^{*}\right)\right),F\left(F^{r}\left(\varkappa_{0}^{*},y_{0}^{*}\right),F^{r}\left(y_{0}^{*},\varkappa_{0}^{*}\right)\right),F\left(F^{r}\left(\varkappa_{0}^{*},y_{0}^{*}\right),F^{r}\left(y_{0}^{*},\varkappa_{0}^{*}\right)\right)\right]\\ \leq \frac{k}{2}[D^{*}\left(F^{r+1}\left(\varkappa_{0}^{*},y_{0}^{*}\right),F^{r}\left(\varkappa_{0}^{*},y_{0}^{*}\right),F^{r}\left(\varkappa_{0}^{*},y_{0}^{*}\right)\right)\\ +D^{*}\left(F^{r+1}\left(y_{0}^{*},\varkappa_{0}^{*}\right),F^{r}\left(y_{0}^{*},\varkappa_{0}^{*}\right),F^{r}\left(y_{0}^{*},\varkappa_{0}^{*}\right)\right)] \end{array}\right\}$$

Utilizing, inequality (3.1), because

$F^{r}\left(\varkappa_{0}^{*},y_{0}^{*}\right)=F\left(\varkappa_{r}^{*},y_{r}^{*}\right)≽\varkappa_{r}^{*}\&F^{r}\left(y_{0}^{*},\varkappa_{0}^{*}\right)=F\left(y_{r}^{*},\varkappa_{r}^{*}\right)≼y_{r}^{*}$ , and because either

$$\begin{array}{c} \varkappa_{r+1}^{*}=F\left(\varkappa_{r}^{*},y_{r}^{*}\right)\neq \varkappa_{r}^{*}\;\text{or}\;y_{r+1}^{*}=F\left(y_{r}^{*},\varkappa_{r}^{*}\right)\neq y_{r}^{*}\\ \leq \frac{k}{2}\left\{\begin{array}{c} \frac{k^{r}}{2}\left[D^{*}\left(F\left(\varkappa_{0}^{*},y_{0}^{*}\right),\varkappa_{0}^{*},\varkappa_{0}^{*}\right)+D^{*}\left(F\left(y_{0}^{*},\varkappa_{0}^{*}\right),y_{0}^{*},y_{0}^{*}\right)\right]\\ +\frac{k^{r}}{2}\left[D^{*}\left(F\left(y_{0}^{*},\varkappa_{0}^{*}\right),y_{0}^{*},y_{0}^{*}\right)+D^{*}\left(F\left(\varkappa_{0}^{*},y_{0}^{*}\right),\varkappa_{0}^{*},\varkappa_{0}^{*}\right)\right] \end{array}\right\} \end{array}$$

Because inequality (3.4) & (3.5) are presumed to hold for s=r , so obtain

$$\leq \frac{k^{r+1}}{2}\left[D^{*}\left(F\left(\varkappa_{0}^{*},y_{0}^{*}\right),\varkappa_{0}^{*},\varkappa_{0}^{*}\right)+D^{*}\left(F\left(y_{0}^{*},\varkappa_{0}^{*}\right),y_{0}^{*},y_{0}^{*}\right)\right].$$

Likewise, we can establish that

$$\left\{\begin{array}{c} \;D^{*}\left(F^{r+2}\left(y_{0}^{*},\varkappa_{0}^{*}\right),F^{r+1}\left(y_{0}^{*},\varkappa_{0}^{*}\right),F^{r+1}\left(y_{0}^{*},\varkappa_{0}^{*}\right)\right)\leq \\ \frac{k^{r+1}}{2}\left[D^{*}\left(F\left(y_{0}^{*},\varkappa_{0}^{*}\right),y_{0}^{*},y_{0}^{*}\right)+D^{*}\left(F\left(\varkappa_{0}^{*},y_{0}^{*}\right),\varkappa_{0}^{*},\varkappa_{0}^{*}\right)\right] \end{array}\right\}$$

In that case, via induction, inequalities (3.4) & (3.5) are verified ∀s≥0 .

In addition, for each positive integer s,r,s<r , we have through the rectangle inequality( $\left(D_{4}^{*}\right)$ of Def-2.1) that

$$\left\{\begin{array}{c} \;D^{*}\left(F^{r}\left(\varkappa_{0}^{*},y_{0}^{*}\right),F^{s}\left(\varkappa_{0}^{*},y_{0}^{*}\right),F^{s}\left(\varkappa_{0}^{*},y_{0}^{*}\right)\right)\leq \\ \;D^{*}\left(F^{r}\left(\varkappa_{0}^{*},y_{0}^{*}\right),F^{r-1}\left(\varkappa_{0}^{*},y_{0}^{*}\right),F^{r-1}\left(\varkappa_{0}^{*},y_{0}^{*}\right)\right)+\\ \;D^{*}\left(F^{r-1}\left(\varkappa_{0}^{*},y_{0}^{*}\right),F^{r-2}\left(\varkappa_{0}^{*},y_{0}^{*}\right),F^{r-2}\left(\varkappa_{0}^{*},y_{0}^{*}\right)\right)\\ +∙∙∙+D^{*}\left(F^{s+1}\left(\varkappa_{0}^{*},y_{0}^{*}\right),F^{s}\left(\varkappa_{0}^{*},y_{0}^{*}\right),F^{s}\left(\varkappa_{0}^{*},y_{0}^{*}\right)\right)\leq \\ \frac{k^{r-1}+∙∙∙+k^{s}}{2}\left[D^{*}\left(F\left(\varkappa_{0}^{*},y_{0}^{*}\right),\varkappa_{0}^{*},\varkappa_{0}^{*}\right)+D^{*}\left(F\left(y_{0}^{*},\varkappa_{0}^{*}\right),y_{0}^{*},y_{0}^{*}\right)\right] \end{array}\right\}$$

Utilizing inequality (3.4)

$$=\left\{\begin{array}{c} \frac{k^{s}\left(1+k+∙∙∙+k^{r-s-1}\right)}{2}\left[D^{*}\left(F\left(\varkappa_{0}^{*},y_{0}^{*}\right),\varkappa_{0}^{*},\varkappa_{0}^{*}\right)+D^{*}\left(F\left(y_{0}^{*},\varkappa_{0}^{*}\right),y_{0}^{*},y_{0}^{*}\right)\right]\\ <\frac{k^{s}}{2\left(1-k\right)}\left[D^{*}\left(F\left(\varkappa_{0}^{*},y_{0}^{*}\right),\varkappa_{0}^{*},\varkappa_{0}^{*}\right)+D^{*}\left(F\left(y_{0}^{*},\varkappa_{0}^{*}\right),y_{0}^{*},y_{0}^{*}\right)\right] \end{array}\right\}$$

This mean, $\;D^{*}\left(\varkappa_{r}^{*},\varkappa_{s}^{*},\varkappa_{s}^{*}\right)<\frac{k^{s}}{2\left(1-k\right)}\left[\;D^{*}\left(\varkappa_{1}^{*},\varkappa_{0}^{*},\varkappa_{0}^{*}\right)+D^{*}\left(y_{1}^{*},y_{0}^{*},y_{0}^{*}\right)\right]$ .

Therefore, $\lim_{r,s\to \infty }\;D^{*}\left(\varkappa_{r}^{*},\varkappa_{s}^{*},\varkappa_{s}^{*}\right)=0$ .

Consequently, utilizing Lemma-2.8, $\left\{\varkappa_{s}^{*}\right\}$ that is, $\left\{F^{s}\left(\varkappa_{0}^{*},y_{0}^{*}\right)\right\}$ is Cauchy sequence and thus is convergent in $D^{*}$ -complete-M-sp X .

$$\text{Assume that}\;\varkappa_{s}^{*}\to \varkappa^{*},\mathrm{as}\;s\to \infty .$$ (3.6)

Likewise, $\left\{y_{s}^{*}\right\},$ mean $\;\left\{F^{s}\left(y_{0}^{*},\varkappa_{0}^{*}\right)\right\}\;$ is as well a Cauchy sequence and consequently is convergent in $\;D^{*}$ -complete-M-sp X .

$$\text{Assume that}\;y_{s}^{*}\to y^{*},\mathrm{as}\;s\to \infty .$$ (3.7)

Next we illustrate that F has (C. F. P) in X .

Utilizing inequalities- (3.4) & (3.6) we obtain

$$\;D^{*}\left(F\left(\varkappa_{s}^{*},y_{s}^{*}\right),\varkappa_{s}^{*},\varkappa_{s}^{*}\right)\leq \frac{k^{s}}{2}\left[\;D^{*}\left(\varkappa_{1}^{*},\varkappa_{0}^{*},\varkappa_{0}^{*}\right)+D^{*}\left(y_{1}^{*},y_{0}^{*},y_{0}^{*}\right)\right].$$

Selecting the limit as s→∞ and utilizing the fact that map F is continuous, we obtain:

$$D^{*}\left(F\left(\varkappa^{*},y^{*}\right),\varkappa^{*},\varkappa^{*}\right)\leq 0,⟹F\left(\varkappa^{*},y^{*}\right)=\varkappa^{*}.$$

Similarly, obtain $F\left(y^{*},\varkappa^{*}\right)=y^{*}$ . Therefore, we verified $\left(\varkappa^{*},y^{*}\right)$ is (C. F. P) of F .

Theorem 3.2: Let $\left(X,D^{*}\right)\;$ be a $D^{*}$ -complete metric space defined on (P.O.S) (X,≼) . Presume that $F:\left(X,D^{*}\right)\times \left(X,D^{*}\right)\to \left(X,D^{*}\right)\;$ satisfying all the conditions in Theorem 3-1, as well as the next conditions:

$$\left(i\right)\;\text{If}\;a\;\text{nondecreasing}\;\left\{\varkappa_{s}^{*}\right\}⟶\varkappa^{*}.\mathrm{So},\varkappa_{s}^{*}≼\varkappa^{*},\forall s,$$ (3.8)

$$\left(\mathrm{ii}\right)\;\text{If}\;a\;\text{nonincreasing}\;\left\{y_{s}^{*}\right\}⟶y^{*}.\mathrm{So},y_{s}^{*}≽y^{*},\forall s.$$ (3.9)

In that case F has (C. F. P).

Proof:
According to the procedures followed in Theorem-3.1, exactly we can reach inequalities (3.6) and (3.7) exactly. So by inequalities- (3.8) & (3.9), we obtain, $\varkappa_{s}^{*}≼\varkappa^{*}\&y_{s}^{*}≽y^{*},\forall s\geq 0$ .

If $\varkappa_{s}^{*}=\varkappa^{*}\;\text{and}\;y_{s}^{*}=y^{*}\;$ for some s, hence, via structures, $\varkappa_{s+1}^{*}=\varkappa^{*}\&y_{s+1}^{*}=y^{*}$ and $\;\left(\varkappa^{*},y^{*}\right)$ is a (C. F. P). As a result we presume either $\varkappa_{s}^{*}\neq \varkappa^{*}\;\text{or}\;y_{s}^{*}\neq y^{*}\;$ .

In that case we obtain

$$\left\{\begin{array}{c} D^{*}\;\left(F\left(\varkappa^{*},y^{*}\right),F\left(\varkappa^{*},y^{*}\right),\varkappa^{*}\right)\leq \\ D^{*}\left(F\left(\varkappa^{*},y^{*}\right),F\left(\varkappa^{*},y^{*}\right),F\left(\varkappa_{s}^{*},y_{s}^{*}\right)\right)+D^{*}\left(F\left(\varkappa^{*},y^{*}\right),F\left(\varkappa^{*},y^{*}\right),\varkappa^{*}\right)\\ =D^{*}\left(F\left(\varkappa_{s}^{*},y_{s}^{*}\right),F\left(\varkappa^{*},y^{*}\right),F\left(\varkappa^{*},y^{*}\right)\right)+D^{*}\left(F\left(\varkappa_{s}^{*},y_{s}^{*}\right),F\left(\varkappa_{s}^{*},y_{s}^{*}\right),\varkappa^{*}\right)\\ \leq \frac{k}{2}\left[D^{*}\left(\varkappa_{s}^{*},\varkappa^{*},\varkappa^{*}\right)+D^{*}\left(y_{s}^{*},y^{*},y^{*}\right)\right]+D^{*}\left(\varkappa_{s+1}^{*},\varkappa_{s+1}^{*},\varkappa^{*}\right)\;\mathrm{by}\;\text{inequality}-\left(3.1\right) \end{array}\right\}$$

Selecting, s→∞ in the above inequality, we get $D^{*}\left(F\left(\varkappa^{*},y^{*}\right),F\left(\varkappa^{*},y^{*}\right),\varkappa^{*}\right)=0,$ this mean $\;F\left(\varkappa^{*},y^{*}\right)=\varkappa^{*}$ . Likewise, we obtain $F\left(y^{*},\varkappa^{*}\right)=y^{*}$ .

Theorem 3.3: Let $\left(X,D^{*}\right)\;$ be $D^{*}$ -complete metric space defined on (P.O.S) (X,≼) . Presume that $F:\left(X,D^{*}\right)\times \left(X,D^{*}\right)\to \left(X,D^{*}\right)$ a continuous map containing the (M.M.P) on X , (s. t) $F\left(\varkappa^{*},y^{*}\right)≼F\left(y^{*},\varkappa^{*}\right)\;$ when $\varkappa^{*}≼y^{*}.$ Suppose that ∃k∈[0,1) (s. t), $\forall \varkappa^{*},y^{*},z^{*},p,q,w^{*}\in X$ , (3.1) holds, when $\varkappa^{*}≽p≽w^{*},y^{*}≼q≼z^{*}\&\varkappa^{*}≺y^{*}$ wherever $p\neq w^{*}\text{or}\;q\neq z^{*}$ .

*If* $\exists \varkappa_{0}^{*},y_{0}^{*}\in X$ , (s. t) $\varkappa_{0}^{*}≼y_{0}^{*},\varkappa_{0}^{*}≼F\left(\varkappa_{0}^{*},y_{0}^{*}\right)\&y_{0}^{*}≽F\left(y_{0}^{*},\varkappa_{0}^{*}\right)$ , so F has (C. F. P) in X .

Proof:
Through the circumstances of above theorem $\exists \varkappa_{0}^{*},y_{0}^{*}\in X\;$ (s. t)

$$\;\varkappa_{0}^{*}≼F\left(\varkappa_{0}^{*},y_{0}^{*}\right)\&y_{0}^{*}≽F\left(y_{0}^{*},\varkappa_{0}^{*}\right).$$

We describe $\varkappa_{1}^{*},y_{1}^{*}\in X\;\mathrm{as}\;\;\varkappa_{1}^{*}=F\left(\varkappa_{0}^{*},y_{0}^{*}\right)≽\varkappa_{0}^{*}\&y_{1}^{*}=F\left(y_{0}^{*},\varkappa_{0}^{*}\right)≼y_{0}^{*}$ .

Because, $\varkappa_{0}^{*}≼y_{0}^{*}$ , we obtain, via circumstances of the theorem, $F\left(\varkappa_{0}^{*},y_{0}^{*}\right)≼F\left(y_{0}^{*},\varkappa_{0}^{*}\right)$ .

For this reason, $\varkappa_{0}^{*}≼\varkappa_{1}^{*}=F\left(\varkappa_{0}^{*},y_{0}^{*}\right)≼F\left(y_{0}^{*},\varkappa_{0}^{*}\right)=y_{1}^{*}≼y_{0}^{*}$ .

Ongoing the above processes we get two sequences $\left\{\varkappa_{s}^{*}\right\}\&\left\{y_{s}^{*}\right\}$ recursively as follows.

$$\forall s\geq 1,\varkappa_{s}^{*}=F\left(\varkappa_{s-1}^{*},y_{s-1}^{*}\right)\&y_{s}^{*}=F\left(y_{s-1}^{*},\varkappa_{s-1}^{*}\right),$$ (3.10)

Such that

$$\;\left\{\begin{array}{c} \varkappa_{0}^{*}≼F\left(\varkappa_{0}^{*},y_{0}^{*}\right)=\varkappa_{1}^{*}≼∙∙∙≼F\left(\varkappa_{s-1}^{*},y_{s-1}^{*}\right)=\varkappa_{s}^{*}≼∙∙∙≼y_{s}^{*}\\ =F\left(y_{s-1}^{*},\varkappa_{s-1}^{*}\right)≼∙∙∙≼y_{1}^{*}=F\left(y_{0}^{*},\varkappa_{0}^{*}\right)≼y_{0}^{*} \end{array}\right\}\;$$ (3.11)

In special, we have ∀s≥0 ,

$$\varkappa_{s}^{*}≼F\left(\varkappa_{s}^{*},y_{s}^{*}\right)=\varkappa_{s+1}^{*}≼y_{s+1}^{*}=F\left(y_{s}^{*},\varkappa_{s}^{*}\right)≼y_{s}^{*}.$$

When, $\varkappa_{s}^{*}=y_{s}^{*}=c$ for some s , in that case c≼F(c,c)≼F(c,c)≼c. It's illustrates, c=F(c,c) . Therefore (c,c) is (C. F. P). Therefore, we assume that

$$\;\varkappa_{s}^{*}≼y_{s}^{*},\forall s\geq 0.$$ (3.12)

Additional, via identical cause as declared in Theorem-3.1, presume $\left(\varkappa_{s}^{*},y_{s}^{*}\right)\neq \left(\varkappa_{s+1}^{*},y_{s+1}^{*}\right)$ .

In that case, in sight of inequality (3.12), ∀s≥0, inequality (3.1) hold and

$$\varkappa^{*}=\varkappa_{s+2}^{*},p=\varkappa_{s+1}^{*},w^{*}=\varkappa_{s}^{*},y^{*}=y_{s}^{*},q=y_{s+1}^{*}\&z^{*}=y_{s+2}^{*}.$$

Rest of evidence is achieved via reiterating similar procedures as in Theorem-3.1.

Theorem 3.4: Let $\left(X,D^{*}\right)\;$ be $D^{*}$ -complete metric space defined on (P.O.S) (X,≼) . Presume that $F:\left(X,D^{*}\right)\times \left(X,D^{*}\right)⟶\left(X,D^{*}\right)\;$ satisfing all the conditions in Theorem-3.3, as well the next conditions:

(i) If nondecreasing $\left\{\varkappa_{s}^{*}\right\}⟶\varkappa^{*}.$ So, $\varkappa_{s}^{*}≼\varkappa^{*},\forall s$ ,
(ii) If nonincreasing $\left\{y_{s}^{*}\right\}⟶y^{*}.$ So, $\;y_{s}^{*}≽y^{*},\forall s$ .

In that case F has (C. F. P).

Proof:
This evidence is straightforward and analogous to that of Theorem-3.2.

Next, discuss the following example, which extends to that of ^21^:

Example 3.5: Suppose that $X={\mathbb{N}}_{0}$ and $\;D^{*}:X^{3}\to \left[0,\infty \right)$ is described as follows:

$$\left\{ \begin{array}{ll} 0 & \text{if}\;\varkappa^{*}=y^{*}=z^{*}\;\\ z^{*}+1 & \text{if}\;\varkappa^{*}=0,y^{*}=0,z^{*}\neq 0\;\\ y^{*}+2 & \text{if}\;\varkappa^{*}=0,y^{*}=z^{*}\neq 0\;\\ y^{*}+z^{*}+1 & \text{if}\;\varkappa^{*}=0,y^{*}\neq z^{*},\left(s.t\right)y^{*},z^{*}\text{diverse of}\;0\;\\ \varkappa^{*}+z^{*} & \text{if}\;\varkappa^{*}=y^{*}\neq z^{*},\text{each diverse of}\;0,\\ \;\varkappa^{*}+y^{*}+z^{*} & \text{if}\;\varkappa^{*},y^{*},z^{*},\mathrm{are}\;\text{each diverse with diverse of}\;0\; \end{array}\;\right.$$

Because, $\;\left(X,D^{*}\right)$ satisfies (iii) in Def. (2.3), in that case $\left(X,D^{*}\right)$ is $D^{*}$ -complete-M-sp. Assume that a (P. O) ≼ described on X as follows: $\forall \varkappa^{*},y^{*}\in X,$ (s. t) $\;\varkappa^{*}≼y^{*}$ holds, if $\varkappa^{*}>y^{*}$ with 0≼1&3≼1 hold.

Assume a map F:X×X→X is described as:

$$F\left(\varkappa^{*},y^{*}\right)=\left\{ \begin{array}{cc} 1 & \text{if}\;\varkappa^{*}≺y^{*}\;\\ 0 & \text{if}\;\text{otherwise}\; \end{array}\right.$$

Presume that, $\;w^{*}≼p≼\varkappa^{*}≺y^{*}≼q≼z^{*}$ hold, so via equivalent form, obtain $w^{*}\geq p\geq \varkappa^{*}>y^{*}\geq q\geq z^{*}$ **.** In that case $F\left(\;\varkappa^{*},y^{*}\right)=F\left(\;p,q\right)=F\left(w^{*},z^{*}\right)=1$ . Consequently , the left-hand side of inequality (3.1) is $D^{*}\left(1,1,1\right)=0$ , thus (3.1) is satisfied.

After that with $\varkappa_{0}^{*}=81\&y_{0}^{*}=0.$ Theorem (3.4) is appropriate for this Example-3.5. It may be viewed in this example that the (C. F. P) is not unique. Therefore, (0, 0) and (1,0) are two (C. F. P) of map F .

The next Remarks are analogy of the Remarks in [21] in $D^{*}$ -M-sp:

Remark 3.6: We observed through Lemma 2.10 that $D^{*}$ -metric induces a metric $d_{D^{*}}$ on X via

$d_{D^{*}}\left(\varkappa^{*},y^{*}\right)=D^{*}\left(\varkappa^{*},y^{*},y^{*}\right)+D^{*}\left(y^{*},\varkappa^{*},\varkappa^{*}\right),\forall \varkappa^{*},y^{*}\in X$ , for $D^{*}$ -Symmetric $d_{D^{*}}\left(\varkappa^{*},y^{*}\right)=2D^{*}\left(\varkappa^{*},y^{*},y^{*}\right),\forall \varkappa^{*},y^{*}\in X$ . Because of the circumstance $p\neq w^{*}\text{or}\;q\neq z^{*},$ inequality- (3.1) doesn’t minimize to any metric inequality with metric $d_{D^{*}}$ . Therefore, our theorems do not minimize fixed point issues analogous to $\left(X,d_{D^{*}}\right)$ .

## 4. Conclusions

The theorems of coupled fixed points in generalized partially ordered metric spaces represent a significant part of confirming the existence and uniqueness of solutions for various integral type equations in pure mathematics and applied sciences such as mathematical models, optimization, control theory, approximation theory, discrete dynamics, and economic theories. Therefore, novel extended categories of coupled fixed point theorems for continuous maps satisfying the property of mixed monotone in the context of extended partially ordered $D^{*}$ -complete-M-sp have been investigated and proven. In addition, to reinforce our major outcomes, a suitable example is provided. Additionally, our major results in Theorems (3.1 and 3.2) are not appropriate for Example-3.5. This is apparent from the fact that inequality (3.1) is not satisfied when $\;w^{*}=p=\varkappa^{*}=y^{*}=3,z^{*}=1\&q=0\;$ . Finally, our main results, which are related to these types of extended coupled fixed point theorems, extend and improve the various outcomes in the literature. We predict that the discoveries in this study will aid scientists in enhancing the research on popularized partially ordered metric spaces to elevate a universal framework for their practical implementation.

## Ethical approval

We would like to inform you that our study does not require any ethical approval.

## Data Availability

No datasets were generated or analyzed during the current study (Our manuscript type does not require data).
