# Supplementary material for: New generalized Trends of Coupled Fixed Point Theorems for Continuous Mappings Satisfying a Property of Mixed Monotone
Source: F1000Res. 2026 Feb 14;14:1362. Originally published 2025 Dec 5. [Version 2] doi: 10.12688/f1000research.172256.2 (PMC12959754; doi:10.12688/f1000research.172256.2)
Supplement: Peer review history

# Reviewer report: Reviewer response for version 2

**DOI:** 10.5256/f1000research.196441.r459041

**Published:** 2026-02-26

**Authors:** Sanjay Kumar Padaliya

**Affiliations:** 1 S.G.R.R. (P.G.) College, Dehradun, Uttarakhand, India

**Comments for Manuscript (New generalized Trends of Coupled Fixed Point Theorems for Continuous Mappings Satisfying a Property of Mixed Monotone)**

  

1. The abstract mentions result under "extended contraction circumstances". These need to be explicitly defined or referred to by specific theorem numbers early on to avoid uncertainty.
2. The manuscript inconsistently uses D^*-metric spaces, partially ordered D^*-metric spaces, and D^ *-complete metric in keywords. Authors must choose correct notation and apply it uniformly throughout the entire manuscript.
3.  Lemma 2.8 requires a more detailed proof to be fully acceptable for technical soundness.
4. Authors must explicitly explain the significance of the inequality in remark 2.12 within a non-D*-symmetric space to satisfy the technical correctness.
5. The current conclusion is vague, stating that "main results... have been explored novel various outcomes". It needs to be rewritten to highlight the specific, concrete mathematical objective achieved.
6. While authors predict the study will aid scientists in "practical implementations", Authors must specify which practical areas are most impacted.
7. It is currently required to determine and explain the relationships between the four theorems presented, rather than just listing them as separate results.
8. Authors need to address whether the major results remain valid if a weaker type of continuity is used, as this is a key point of interest for improving the paper's scope.
9.  Correct the use of "uncommon forms" and English throughout the manuscript (e.g., phrases like "After witnessing the implementations..."). Also check for spelling mistakes throughout the manuscript (for example it is “Hausdorff” not “Housdorff” in remark 2.4.)
10. Remove repetitive words, such as the multiple uses of "various" in the same sentence in the abstract and conclusion.

If authors address above points the manuscript **can be indexed.**

Is the work clearly and accurately presented and does it cite the current literature?

Yes

If applicable, is the statistical analysis and its interpretation appropriate?

No

Are all the source data underlying the results available to ensure full reproducibility?

Yes

Is the study design appropriate and is the work technically sound?

Yes

Are the conclusions drawn adequately supported by the results?

Yes

Are sufficient details of methods and analysis provided to allow replication by others?

Yes

Reviewer Expertise:

Fixed Point Theory, Fuzzy Analysis and Fractal Geometry.

I confirm that I have read this submission and believe that I have an appropriate level of expertise to confirm that it is of an acceptable scientific standard, however I have significant reservations, as outlined above.

# Reviewer report: Reviewer response for version 2

**DOI:** 10.5256/f1000research.196441.r458567

**Published:** 2026-02-24

**Authors:** Rahim Shah

**Affiliations:** 1 Kohsar University Murree, Murree, Pakistan

The suggested revisions have been completed, and the paper is now suitable for acceptance for indexing.

Is the work clearly and accurately presented and does it cite the current literature?

Yes

If applicable, is the statistical analysis and its interpretation appropriate?

Not applicable

Are all the source data underlying the results available to ensure full reproducibility?

No source data required

Is the study design appropriate and is the work technically sound?

Yes

Are the conclusions drawn adequately supported by the results?

Yes

Are sufficient details of methods and analysis provided to allow replication by others?

Yes

Reviewer Expertise:

Analysis, Fixed Point Theory, Applied Mathematics

I confirm that I have read this submission and believe that I have an appropriate level of expertise to confirm that it is of an acceptable scientific standard.

# Reviewer report: Reviewer response for version 1

**DOI:** 10.5256/f1000research.189971.r440891

**Published:** 2026-01-12

**Authors:** Jamil Mahmoud

**Affiliations:** 1 University of Diyala, Diyala, Iraq

**Comments for** **Manuscript** **(New generalized Trends of Coupled Fixed Point Theorems for Continuous Mappings Satisfying a Property of Mixed Monotone)**

  

1. Uniqueness: which conditions must be met for a continuous mapping to have a unique double fixed point that satisfies the mixed monotone property.

 

1. Relationships: the authors offered three details proofs for each of the four theorems. it is essential to determine their relationships and give examples support their arguments.

 

1. The major results that authors presented in this paper can be valid when we deploy a weaker type of continuity.

 

1. Conclusions: the conclusion that given by authors can be summarized in the following: first, the applications of coupled fixed point in generalized partially ordered metric space. Second, viewing an extended category of couple fixed point theorems for a continuous map satisfying the mixed monotone property. Third, the authors claim that these results will help future researchers for practical implementation. The article's main objective should be highlighted in the conclusion, along with a brief explanation of how it would benefit them. The conclusion section needs to be more specific.

 

1. Linguistic issues: Although the article was written in good English overall, the authors committed a few mistakes, such as utilizing an uncommon form.

 

1. Definition 2.1 ( *D* *3* *)* (Symmetry) where P is permutation map. Since both have the same meaning, there is no need for both. Use P as permutation map.

 

1. Lemma 2.8: giving a detailed proof.

 

1. Lemma 2.10 should be put in this way ( *D* * -symmetric, *d* *D* * *x* * *,* *y* * *=2* *D* * *x* * *,* *y* * *,* *y* *  ).

 

1. Remark 2.12: it stated that *X,* *D* *  is non *D* * -symmetric space, then *D* * -metric properties demonstrate that *3* *2* *D* * *x* * *,* *y* * *,* *y* * *≤* *3D* * *x* * *,* *y* * *,* *y* *  ( is this a required result? Explain).

 I suggest that this work be accepted since it introduces and establishes new generalized results of linked fixed-point theorems for continuous functions with the property of mixed monotone in the context of partially ordered *D* * -complete metric spaces. Additionally, I advise authors to evaluate and address the submitted comments.

 Best wishes

 Your sincerely

 Assist. Prof. Dr. Jamil Mahmoud Jamil

Is the work clearly and accurately presented and does it cite the current literature?

Yes

If applicable, is the statistical analysis and its interpretation appropriate?

Partly

Are all the source data underlying the results available to ensure full reproducibility?

Yes

Is the study design appropriate and is the work technically sound?

Yes

Are the conclusions drawn adequately supported by the results?

Yes

Are sufficient details of methods and analysis provided to allow replication by others?

Yes

Reviewer Expertise:

Main fields of research: topology : fuzzy topology, soft topology, generalized topology, fuzzy Algebra , advanced functional analysis

I confirm that I have read this submission and believe that I have an appropriate level of expertise to confirm that it is of an acceptable scientific standard.

# Response

**Published:** 2026-01-27

**Authors:** Alaa AL-Jumaili

**Affiliations:** Department of mathematics, College of Education for pure sciences, University of Anbar, Ramadi, Alanbar, Iraq

Dear Prof...

 Best greetings.....

 I hope this message finds you well.

 Subject \ comments responses about the manuscript-f1000res172256

  (New generalized Trends of Coupled Fixed Point Theorems for Continuous Mappings Satisfying a Property of Mixed Monotone)-Manuscript Number: 3b619270-4ddc-4685-af42-071e2dbdcc63-f1000res172256)

 First: We extend our sincere thanks to the esteemed evaluator for what he said regarding the paper being good and its results being original, as well as the originality of the examples and applications presented in it.      

 The following is the response to the comments kindly provided by the esteemed resident, and they have been carefully considered due to their importance.

 1- The condition for the uniqueness of a continuous mapping to have a unique double fixed point that satisfies the mixed monotone property is explained in the submitted manuscript.

 2- The relationships between the theorems presented in the research paper are evident from the results obtained through their proofs.

 3- Yes, surely, thank you for your feedback.

 4- The conclusion of the research paper was summarized according to the honored reviewer's instructions.

 5- The uncommon wording in the research paper, as noted by the honored reviewer in his comments, has been corrected.

 6- The definition [2.1] is as stated in the main reference.

 7- Researchers can access the complete proof of the Lemma 2.8 by referring to its reference [4].

 8- Ok, Thank you for all the feedback.

 9- Yes, it is required result.

 Acknowledgments: Thankful to reviewers for their valuable corrections and important suggestions  

# Reviewer report: Reviewer response for version 1

**DOI:** 10.5256/f1000research.189971.r440893

**Published:** 2025-12-31

**Authors:** Rahim Shah

**Affiliations:** 1 Kohsar University Murree, Murree, Pakistan

The paper is well written and technically sound. To further improve its quality, the following minor revisions are recommended. After addressing these comments, the paper may be considered for acceptance for indexing.

1. Rewrite the **Abstract (Background)** in a standard and structured form.
2. Carefully review the manuscript to ensure grammatical correctness throughout.
3. To strengthen the **Introduction** section, the authors should include additional relevant references, such as:
  - Li, et al., 2017 - Ref 1
  - Shah, et al., 2016 - Ref 2
  - George, et al., 2022 - Ref 3

Is the work clearly and accurately presented and does it cite the current literature?

Yes

If applicable, is the statistical analysis and its interpretation appropriate?

Not applicable

Are all the source data underlying the results available to ensure full reproducibility?

No source data required

Is the study design appropriate and is the work technically sound?

Yes

Are the conclusions drawn adequately supported by the results?

Yes

Are sufficient details of methods and analysis provided to allow replication by others?

Yes

Reviewer Expertise:

Analysis, Fixed Point Theory, Applied Mathematics

I confirm that I have read this submission and believe that I have an appropriate level of expertise to confirm that it is of an acceptable scientific standard, however I have significant reservations, as outlined above.

## References

[1] : Fixed point theorems in dislocated quasi-metric spaces. *The Journal of Nonlinear Sciences and Applications* .2017;10(09) : 10.22436/jnsa.010.09.12 4695-4703 10.22436/jnsa.010.09.12

[2] : Some Fixed Point Theorems of Integral Type Contraction in Cone b-metric Spaces. *Turkish Journal of Analysis and Number Theory* .2016;3(6) : 10.12691/tjant-3-6-5 165-169 10.12691/tjant-3-6-5

[3] : Some Fixed Points Results in-Metric and Quasi-Metric Spaces. *Journal of Function Spaces* .2022;2022: 10.1155/2022/1803348 1-6 10.1155/2022/1803348
